# Supplementary material for: The Nebulous Association between Cognitive Impairment and Falls in Older Adults: A Systematic Review of the Literature
Source: Int J Environ Res Public Health. 2023 Feb 1;20(3):2628. doi: 10.3390/ijerph20032628 (PMC9915123; doi:10.3390/ijerph20032628)
Supplement: Supplementary file 1 [file ijerph-20-02628-s001.zip › ijerph-2120947-supplementary.pdf]

How robust is the association between MCI and falls in older adults ? A systematic review

Supplementary data

Search Strategy

Research question:

1. Is there an association between falls and cognitive impairment in older people?
2. What are the underlying mechanisms between them?
3. Are there any efficient strategy and recommendation to prevent falls in cognitively impaired older people?

Published date:
From January 2011 to August 2021
Because there was already a paper from Muir et al in 2012 for the same topic
(doi: 10.1093/ageing/afs012)

Inclusion criteria:
Older people (aged more than 65 years old)
Original studies
All languages accepted

Exclusion criteria:
Review, conference abstract, editorial

Target databases:
Embase, Web of science, Medline, CINAHL (+/- google scholar)

Keywords that we chose for now (maybe there are others?)
For Falls: “falls” OR “fall” OR “falling” OR “injury” OR “gait” OR “gait disorders” OR “balance” OR “gait impairment” OR “balance impairment” OR “fall risk” OR “accidental falls” OR “falling*”
For Cognitive impairment: “cognitive impairment” OR "dementia" OR "mild cognitive impairment" OR "Alzheimer*" OR "vascular dementia" OR “lewy bodies*” OR “lewy body” OR “vascular dementia” OR “cognitive defect*” OR “cognitive dysfunction” OR “parkinson disease” OR “parkinsonism”
For Older people: “older adult*” OR “older people” OR “aged” OR “elderly” OR “oldest old” OR “older one*” OR “over 85” OR “over 65” OR “aged patient*” OR “geriatric patient*” OR “aged people” OR “aged person” OR “aged subject” OR “elderly” OR “elderly patient” OR “elderly people” OR “elderly person” OR “elderly subject” OR “senior citizen” OR “senium”
